# Genomic and Proteomic Characterization of Bacteriophage BH1 Spontaneously Released from Probiotic *Lactobacillus rhamnosus* Pen

**DOI:** 10.3390/v11121163

**Published:** 2019-12-16

**Authors:** Piotr Jarocki, Elwira Komoń-Janczara, Marcin Podleśny, Oleksandr Kholiavskyi, Monika Pytka, Monika Kordowska-Wiater

**Affiliations:** 1 Department of Biotechnology, Microbiology and Human Nutrition, University of Life Sciences in Lublin, 8 Skromna St., 20-704 Lublin, Poland; 2 Process and Development Department, Al. Tysiąclecia Państwa Polskiego 13, Grupa Azoty Zakłady Azotowe “Puławy” S.A, 24-110 Puławy, Poland

**Keywords:** *L. rhamnosus*, probiotics, bacteriophage, spontaneous prophage induction

## Abstract

*Lactobacillus rhamnosus* Pen is a human endogenous strain used for the production of probiotic formula, which is effective in the prevention of antibiotic-associated diarrhoea. Our study showed that this probiotic strain releases bacteriophage BH1 without the addition of any inducing agent. Our research revealed that phage BH1 has a circular genome with a length of 40721 nt and a GC content of 44.8%. The genome of phage BH1 possesses 57 open reading frames which could be divided into functional modules associated with DNA packaging, morphogenesis, lysis, integration, genetic switch, and replication. In spite of similarity in morphology and genomic organization, comparative analysis revealed substantial genetic diversity and mosaic genomic architecture among phages described for the *Lactobacillus casei* group. Additionally, qPCR and ddPCR analysis confirmed earlier microscopic observations indicating that *L. rhamnosus* Pen liberates bacteriophage particles during growth. This occurs spontaneously, and is not a result of external inducing factors. For samples collected after 4 and 24 h of *L. rhamnosus* Pen culture, the number of *att*B and *att*P copies increased 2.5 and 12 times, respectively. This phenomenon, by introducing resistance to other phages or enhancing the biofilm-forming capabilities, may increase the survivability of microorganisms in their natural ecological niche. Conversely, spontaneous phage induction may be an important virulence factor for bacteria, posing a potential threat for the human host.

## 1. Introduction

Human intestines form an ecosystem in which many species of bacteria reside. The presence of some species stabilizes the digestive system, in part by preventing various pathological conditions. Such strains of microbes with health-promoting characteristics are collectively called probiotics. This group of bacteria includes various strains of the genus *Lactobacillus*, which are used intensively in the dairy and pharmaceutical industries due to their health-promoting properties, which are increasingly supported by research [1,2]. One example of commercial health-promoting bacteria is *Lactobacillus rhamnosus* Pen, which is a component of a pharmaceutical used to alleviate diarrhoea associated with antibiotic therapy [3]. It is commonly believed that probiotic bacteria stabilize intestinal microflora, inhibit the growth of pathogenic microbes, eliminate or reduce symptoms of lactose intolerance, prevent or alleviate bacterial and viral diarrhoea, and normalize intestinal motility disorders. It has also been shown that enrichment of the human diet with probiotic bacteria may stimulate the immune system and positively influence the regulation of blood cholesterol levels [4,5].

However, it remains important to conduct further studies on the safety of using bacteria as dietary supplements, as there are also reports of negative features of some microorganisms considered to be probiotics [6,7]. Additionally, prophage sequences are widespread in genomes of *Lactobacillus* bacteria [8,9]. In general, the presence of sequences encoding bacteriophage proteins in the genome was considered a negative phenomenon. Often, their presence is connected with a high probability of induction and release of bacteriophage particles through cell lysis. Unsurprisingly, such processes result in large losses in the food industry, where bacteria of the genus *Lactobacillus* are very widely applied [10,11]. Interestingly, scientific studies indicate that the presence of prophage sequences in bacterial genomes not only increases the genetic variability, but may also have a positive effect on the bacterial host. Therefore, prophage sequences cannot be treated only as a potential threat to bacterial cells, which would undergo lysis at the time of activation of prophages and their transition to the lytic cycle. The presence of prophages in bacterial genomes requires further analysis, taking into account their positive effects, such as increasing the viability of bacterial cells and enabling them to gain an environmental advantage in a given ecological niche [12,13].

The analysis of genome sequences publicly available in bioinformatic databases indicates that, despite high affinity, the bacteria belonging to the *L. casei* group (*L. casei*, *L. paracasei,* and *L. rhamnosus*) possess substantially diversified sequences of phage origin in their genomes. Furthermore, results of studies also conducted by our research group suggest that bacteriophages associated with the above species of intestinal bacteria are characterized by various forms of existence in genomes of these microorganisms. A good example in this case may be the lytic phage Lc-Nu [14] or bacteriophage Lrm1, described by Durmaz et al. [15] which, due to the lack of lytic activity against different strains of *L. rhamnosus*, was classified as defective. We also demonstrated that BH1 phage has a diverse nature. The analysed probiotic strain — *L. rhamnosus* Pen-released high amounts of this bacteriophage during culture growth, and interestingly, this phenomenon did not result from the application of an external inducing agent [16]. There is a potential physiological importance of such a co-existence of the phage and the bacterium; some researchers suggest that the phage may bring potential benefits for the bacterial host, and could play an important role in bacterial virulence [17].

In this study, we carried out genomic and proteomic characterization of bacteriophage BH1 isolated from probiotic *Lactobacillus rhamnosus* Pen. Additionally, the process of spontaneous prophage induction (SPI) during bacterial growth was monitored using qPCR and droplet digital PCR method. Finally, the potential physiological significance of SPI for bacteria and their host was analysed and briefly discussed.

## 2. Materials and Methods

### 2.1. Bacterial Strains and Culture Conditions

*Lactobacillus rhamnosus* Pen obtained from Biomed Serum and Vaccine Production Plant Ltd. in Lublin (Poland) was routinely cultured anaerobically in a Man-Rogosa-Sharpe broth (Difco) at 37 °C. For prophage induction, 1 mL of an overnight culture was added to 100 mL of MRS with 10 mM CaCl_2_ and then when OD_600_ reached 0.2, mitomycin C was supplemented at a final concentration of 0.5 µg/mL. Next, the culture was incubated at 37 °C for 18 h, and afterwards lysate was centrifuged (5000× *g*, 10 min, 4 °C) and filtered using 0.45 pore diameter membranes. Phage particles were precipitated by the addition of 10% (*w*/*v*) PEG 8000 and 0.5 M NaCl (overnight, at 4 °C). After centrifugation (10,000× *g*, 1 h, 4 °C), the phages were resuspended in a 1 ml SM buffer. The residual PEG was removed by extraction with chloroform [18].

### 2.2. Electron Microscopy

Phage particles were fixed on formvar carbon-coated copper grids and stained negatively with 1% (w/v) phosphotungstic acid. Then, bacteriophages were imaged on LEO 912AB transmission electron microscope (LEO Electron Microscopy Inc., Thornwood, NY, USA).

### 2.3. Protein Identification

For electrophoresis (SDS-PAGE, 2DE), the protein samples were prepared with a 2D Clean-Up kit (GE Healthcare, Chicago, IL, USA) and separated on 7 cm linear IPG strips (pH 3–10) using Protean IEF (Bio-Rad, Hercules, CA, USA) and mini Protean Tetra cell (Bio-Rad) with 4%–20% precast polyacrylamide gels. Selected protein bands were excised from the gel and analysed by liquid chromatography coupled to the mass spectrometer in the Laboratory of Mass Spectrometry, Institute of Biochemistry and Biophysics, Polish Academy of Sciences (Warsaw, Poland). Samples were concentrated and desalted on a RP-C18 precolumn (Waters, Milford, MA, USA), and further peptide separation was achieved on a nano-ultra performance liquid chromatography (UPLC) RP-C18 column (Waters, BEH130 C18 column, 75 µm i.d., 250 mm long) of a nanoACQUITY UPLC system, using a 45 min linear acetonitrile gradient. Column outlet was directly coupled to the electrospray ionization (ESI) ion source of the Orbitrap Velos type mass spectrometer (Thermo, Waltham, MA, USA), working in the regime of data dependent MS to MS/MS switch with HCD type peptide fragmentation. An electrospray voltage of 1.5 kV was used. Raw data files were pre-processed with the Mascot Distiller software (version 2.4.2.0, MatrixScience, Boston, MA, USA). The obtained peptide masses and fragmentation spectra were matched to the National Center Biotechnology Information (NCBI) nonredundant database, with a bacteria filter using the Mascot search engine (Mascot Daemon v. 2.4.0, Mascot Server v. 2.4.1, MatrixScience, Boston, MA, USA). The following search parameters were applied: Enzyme specificity was set to trypsin, peptide mass tolerance to ± 20 ppm, and fragment mass tolerance to ± 0.1 Da. The protein mass was left as unrestricted, and mass values as monoisotopic, with one missed cleavage being allowed. Alkylation of cysteine by carbamidomethylation as fixed, oxidation of methionine was set as a variable modification.

Protein identification was performed using the Mascot search engine (MatrixScience, Boston, MA, USA), with the probability-based algorithm. The expected value threshold of 0.05 was used for analysis, which means that all peptide identifications had less than 1 in 20 chance of being a random match.

### 2.4. Genomic Analysis

Phage DNA was prepared using Virus Mini AX transfect kit (A&A Biotechnology, Gdansk, Poland) according to the manufacturer’s instructions. Genome sequencing was carried out at the Genomed using an Illumina MiSeq 250 bp paired-end run with a 300 bp insert library (Illumina, San Diego, CA, USA). The raw reads were trimmed and de novo assembled using CLC Genomics Workbench (Qiagen, Hilden, Germany). The assembly was verified by Sanger sequencing. The complete sequence was annotated by using Prokka tool v. 1.12 (Victorian Bioinformatics Consortium, Melbourne, Australia) and the identified ORFs were analysed by BLASTp (NCBI, Bethesda, MD, USA) and InterProScan (EMBL-EBI, Hinxton, UK). The following programs were used to analyse the obtained BH1 sequence: Easyfig version 2.2.3, PhagTerm version 1.0.12, Signal IP version 5.0, MegaBlast (NCBI, Bethesda, MD, USA) and CLC Sequence Viewer 8 (Qiagen, Hilden, Germany). The genome sequence of BH1 has been deposited in the GenBank database under accession number MH983004.1.

### 2.5. Analysis of Prophage Induction—Sample Preparation

In an experiment aiming to examine the BH1 phage induction using mitomycin C, *L. rhamnosus* Pen was cultured in a MRS medium (vol. 10 mL) at 37 °C, until OD_600_ reached the value of 0.5. The bacteria were then centrifuged (7000× *g*, 5 min) and suspended in fresh MRS medium. Subsequently, a specific amount of inducer was added to the culture (final concentration of mitomycin was 0.25, 0.5, 1 and 2 µg/mL). The control sample consisted of cultures not treated with an inducer. After 6 h of induction, OD_600_ was measured and 2 mL of each culture was collected. The samples were centrifuged for 5 min at 7000× *g*, after which the cell deposit was frozen at 20 °C. The experiment was performed in three biological replicates.

In the case of spontaneous prophage induction (SPI), 50 mL of MRS medium was inoculated with an initial culture at a ratio of 1:100. Then, every 4 h (after 4, 8, 12, 16, 20, and 24 h, respectively), the optical density was measured and samples for DNA isolation were collected. In this case, the control samples were those collected after 8 h. Samples were prepared as described above. The experiment was conducted in five biological replicates.

After thawing, bacterial deposits were used to isolate total DNA with the genomic mini AX bacteria+ kit from A&A Biotechnology. The isolation was performed in accordance with the maker’s recommendations, and the concentration of nucleic acids was estimated using the Nanodrop 2000c spectrophotometer (Thermo, Waltham, MA, USA). The obtained DNA samples were subsequently used for quantitative analysis of *att*P, *att*B, *att*L, and *att*R sequences by qPCR and droplet digital PCR methods.

### 2.6. Quantitative PCR and Droplet Digital PCR

The quantitative analysis of the excision of the prophage from *L. rhamnosus* Pen genome required designing four pairs of primers flanking the integration loci of the phage BH1 (*att*P, *att*B, *att*L, and *att*R) and one pair of primers (Lrh) that generated the reference product (total bacteria count) (Table 1) (Figure 3). Real-time PCRs were performed in 15 µL with the use of a SG qPCR master mix (Eurx, Gdansk, Poland) reagents on the CFX apparatus from Biorad (Hercules, CA, USA). In the reaction, primers with a final concentration of 0.3 µM and appropriately diluted DNA solutions were used. qPCR was conducted under conditions recommended by the reagent manufacturer (predenaturation at 95 °C, 3 min, denaturation at 94 °C, 15 s; annealing at 60 °C, 30 s; elongation at 72 °C, 30 s). At the end of the reaction, to confirm the specificity of amplification, the melting curves of the obtained PCR products were analysed. The results were analysed with the use of CFX Manager v. 3.1 software (Biorad, Hercules, CA, USA), taking into account the yield of reaction [19].

Reactions for droplet digital (ddPCR) were prepared in 20 µL volumes with 10 μL QX200 ddPCR EvaGreen Supermix, 2 μL of 1 μM forward and reverse primers, and 1 µL of 10 or 100× water-diluted DNA. As a no template control (NTC) 1 μL of water was used. The 20 μL droplet digital PCR reaction mixture was then loaded into the disposable droplet generator cartridge (DG8 cartridge, Biorad). 70 μL of droplet generation oil was loaded into the oil well for each sample. The cartridge was placed into the droplet generator (QX100 Droplet Generator, Biorad). The generated droplets were transferred to 96-well reaction plate (ddPCR plater 96-well, Semi-Skirted, Biorad). The plate was heat-sealed with a sealer (PX1 PCR Plate Sealer, Biorad). Then, the plate was placed on a thermal cycler (T100 Thermal Cycler, Biorad) and amplified to the endpoint. Thermal-cycling conditions were 95 °C × 10 min (1 cycle), 95 °C × 30 s (ramp rate 2 °C/s), and 55 °C × 60 s (ramp rate 2 °C/s) (40 cycles), 98 °C × 10 min (1 cycle), and a 12 °C hold. Following PCR amplification, the 96-well PCR plate was loaded on a droplet reader (QX200 Droplet Reader, Biorad), which reads the droplets from each well of the plate. Analysis of the ddPCR data was performed with the QuantaSoft analysis software version 1.7.4.0917. Results with droplets number >10,000 were analysed.

### 2.7. Statistical Analysis

The data from qPCR and ddPCR were analysed by the Microsoft Excel 2016 (Microsoft Corporation, Redmond, WA, USA) and the Statistica version 13.3 (StatSoft, Tulsa, OK, USA) using ANOVA procedure for analysis of variance. *p* < 0.05 was considered statistically significant.

## 3. Results and Discussion

### 3.1. Morphological and Proteomic Characterization of Bacteriophage BH1

**Figure 1:**
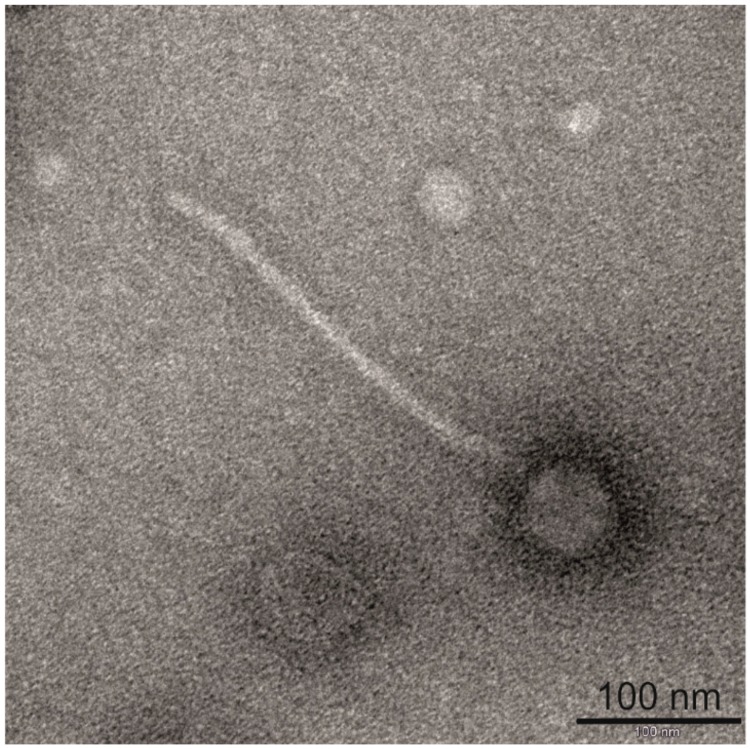
Transmission electron microscope micrograph of bacteriophage BH1 released by the *L. rhamnosus* strain Pen.

The spontaneous releasing of bacteriophage particles by *L. rhamnosus* Pen has been observed in previous work. This phenomenon did not require any inducing agent, and did not significantly affect bacterial growth. Additionally, similar to phage Lrm1, the propagating host for BH1 was not identified in the standard experimental conditions [15,16]. In this work, we carried out a thorough characterization of BH1 bacteriophage. Phage particles were obtained using standard procedures, in which mitomycin C-induced cultures were concentrated using polyethylene glycol. The final samples were used to take photographs with a transmission electron microscope (Figure 1). Our observations confirmed that these methods successfully isolated complete phage particles. The measurements showed that the BH1 bacteriophage particles had tails approximately 287 nm long and isometric heads approximately 58 nm in diameter. As in the case of phage Lrm1, the morphology indicated that the bacteriophage BH1 can be classified in the *Siphoviridae* family [15,20].

**Figure 2:**
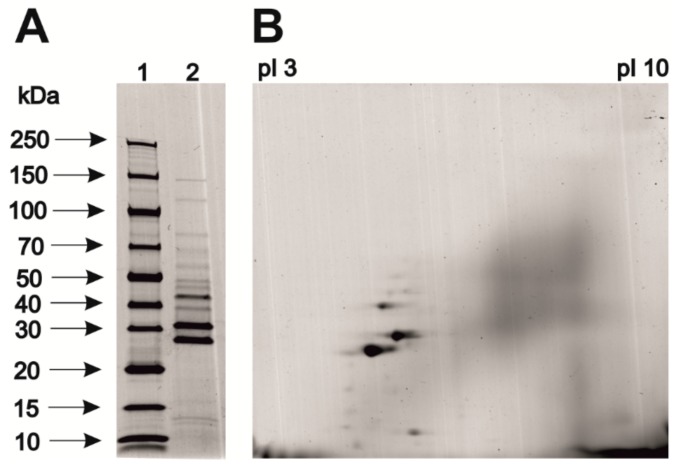
Electrophoretic separation of bacteriophage-derived proteins. Analysis was conducted using SDS-PAGE (**A**) and two-dimensional electrophoresis procedure (**B**).

Next, phage samples were concentrated and prepared for protein electrophoretic examination (SDS-PAGE and 2DE—Figure 2). The resultant electrophoretic profiles of the analysed protein sample showed numerous protein bands, which were excised from the gels. For protein identification, the samples were digested with trypsin and then peptide mixtures were analysed by nano-HPLC coupled to an ESI-orbitrap spectrometer.

Among the detected proteins were the following phage-derived structural polypeptides: Tail proteins, phage tail tape measure protein, capsid protein, portal protein, and head-tail adapter protein. Apart from typical structural proteins, peptides derived from proteins performing other functions were also identified, such as DNA-packaging protein, holin, transcriptional regulator, and peptidase U35 (Table 2). As well as proteins derived from phage, bacterial proteins were also detected. These proteins could represent impurities in the samples, as these contained bacteriophages, as well as bacterial host secreted proteins. However, in some cases, identified bacterial proteins may play an important role in the composition of mature phage particles. For example, the proteins GroEL and GroES, which belong to the chaperonin family, are essential for the correct assembly of bacteriophage tails and heads [21].

### 3.2. Bacteriophage BH1 Genome Analysis

Next, the bacteriophage particles were used for DNA isolation, and complete genomic sequence of BH1 was determined using an Illumina MiSeq system (a total of 147,084 reads with mean read length of 236.37 bp resulting in 848.73-fold average coverage of analysed genome). The obtained sequence was verified using Sanger sequencing. The final assembled consensus sequence revealed a circular, double stranded DNA genome with a size of 40,721 bp. The total length of the BH1 sequence was similar to previously described temperate phage Lrm1 isolated from industrial *L. rhamnosus* M1 (39,989 bp) [15]. Interestingly, the complete genome of the virulent phage Lc-Nu of probiotic *L. rhamnosus* strain Lc 1/3 was ~4.5 kb shorter. This difference was due to the lack of some lysogenic regions similar to integrase coding gene or parts of sequences such as *att*P and CI-like lytic cycle repressor, which are associated with virulence of Lc-Nu bacteriophage [14]. The G + C content of BH1 genome was 44.8%; very close to previously described bacteriophages for the *Lactobacillus casei* group: Lc-Nu (44.3%), ϕAT3 (44.6%), A2 (44.9%), Lca1 (44.8%), and Lrm1 (45.5%). The G + C content also corresponded to the overall GC-content determined for chromosomal DNA of bacterial host of BH1—*L. rhamnosus* Pen (46.8%), and for other previously reported *L. rhamnosus* strains (average value of 46.7%) [22,23,24].

**Figure 3:**
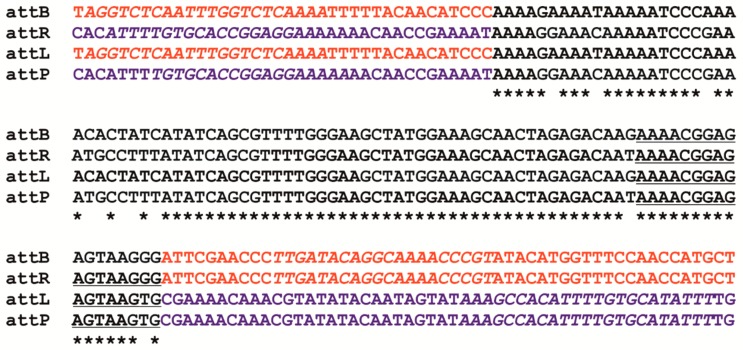
Alignment of phage attachment sites derived from phage BH1 and *L. rhamnosus* Pen genome. The genome sequence of *L. rhamnosus* was marked in red; the phage sequence/prophage sequence in blue; the core sequences are underlined; the motifs used to design the primers (Table 1) were highlighted in italics.

The analysis of raw data from NGS sequencing using PhageTerm showed the presence of—5′-CGATCGACCT-3′ cos sequences similar to those previously determined for bacteriophages produced by other strains belong to the *L. casei* group. However, two mismatches within the cos sequence of BH1 were also detected [15]. The alignment of *att*P sequences showed that the BH1 phage attachment site is localized between ORF22 and ORF23, adjacent to integrase coding gene. The attachment site of BH1 consists of a 90-nucleotide sequence with 15-nt core (Figure 3), showing 87.5% similarity with phage Lrm1, and 96% similarity with Lc-Nu bacteriophage (with only 58% sequence coverage), seems to be characteristic of this phage.

**Figure 4:**
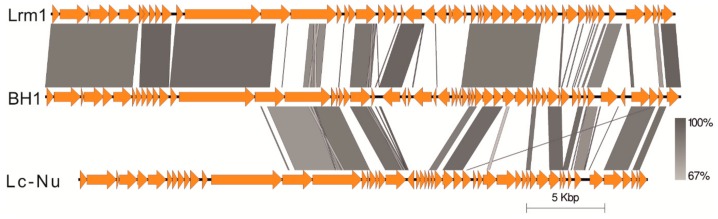
Visualization of genome maps alignment of three *Lactobacillus rhamnos*us bacteriophages—BH1, Lrm1, and Lc-Nu. Putative open frames are marked with arrows. Lines represent the level of homology between analyzed phage sequences (BLAST identity is presented in gray scale).

In general, genome sequence of ϕBH1 showed the highest similarity with prophage sequences detected in genomes of several *L. rhamnosus* strains: CLS17, Lrh10, DS3, DS9, DS12, DS18, ASCC 3029, R0011, B9, ARJD, IBL027, Lr032, and ATCC 21052 (Dataset S2). A lower level of homology was revealed with two *L. rhamnosus* phages—Lrm1 (NC_011104.1, identity 94%, query coverage 59%) and Lc-Nu (NC_007501.1, identity 91%, query coverage 28%) (Figure 4) and other bacteriophages and prophages described for the *L. casei* group (Dataset S1, S3, S4). These findings, in conjunction with other reports, suggest a high diversity of *L. rhamnosus* phage genomic sequences, highlighting that sequences of a bacteriophage origin may be a very interesting source of information regarding bacteria phylogenetics [9,25]. Moreover, our results also confirm earlier works by Brandt and Allatosava [26] and Zago et al. [27], demonstrating that phage sequences have a high potential for the identification of bacteria of the genus *Lactobacillu*s, even at the strain level. Interestingly, in genomes of many *L. rhamnosus* strains, short, approximately 30 nucleotide sequences were detected, which were either identical or very similar to the sequences observed in the BH1 genome. These sequences were located within the CRISPR (clustered regularly interspaced short palindromic repeat) modules, indicated by both flanking sequences and the proximity of genes encoding Cas endonuclease and type II-A CRISPR-associated Csn2 proteins. The presence of phage sequences within the CRISPR modules likely makes these bacterial strains resistant to infection caused by the BH1 bacteriophage. Previous studies have shown that this phenomenon is a highly specific, sequence-dependent defense system against phage infections [25,28].

### 3.3. Genome Organization of Bacteriophage BH1

Phage BH1 has a genome organization typical for other *Lactobacillus* bacteriophages with the following modules: Packaging, structural proteins, lysis, integration, genetic switch, and replication. Fifty-seven open reading frames (fifty genes on the positive strain and seven genes on the negative strand) were predicted, which harboured nearly 91.4% of the whole sequence determined. The ORF’s GC-content ranged from 35.2% (ORF24) to 50.8% (ORF7). Regarding the start codon, forty-four ORFs start with ATG, eight with GTG, and five genes had the TTG start codon. Among those revealed, most ORFs were preceded by atypical Shine-Delgarno sequence, complementary to the 3′ end of the 16S rRNA gene of *Lactobacillus* species (3′-UCCUCCAA-5′) [29]. The location of predicted ORFs and their putative functions are presented in Table 3.

Comparative studies revealed that ORF1 and ORF2 correspond to the terminase small and large subunits, respectively. Presumably, these two terminase subunits are responsible for specific DNA binding (HTH-domain), and cutting concatemeric DNA into genome lengths. Therefore, they most likely represent the bacteriophage DNA packaging module [30]. Similar to phage Lrm1 and *L. casei* phage A2, ORF57 encodes HNH endonuclease, which may be also involved in the DNA packaging system. Since Garcia et al. [31] showed that this HNH protein can be also classified as a phage terminase (small subunit), we speculated that the predicted protein products of BH1 ORF1 and ORF57 likely have redundant activity.

As in the case of phage Lrm1, ORF3 encodes a 63-amino-acid putative integral membrane protein, possessing two transmembrane-spanning domains. The next three ORFs (ORF4, ORF5, and ORF6) are involved in head morphogenesis. The products of these genes—portal protein, head maturation protease, and major head protein (with a CCCAAAA slippery sequence)—show an amino acid identity of about 90% with bacteriophage Lrm1 (91.9%, 94.3%, and 89.3%, respectively), and also high aa similarity with three other *L. casei* group phages—J-1, PL-1, and A2 [15,31,32]. ORFs 8 to 11 constitute a typical neck region consisting of DNA-packaging protein, phage head-tail adapter, head-tail joining protein, and phage-related head-to-tail joining protein. Interestingly, we observed an additional open reading frame (ORF7) between the head and neck module, not detected in the genome of Lrm1. This gene encodes a hypothetical protein containing putative Ig-like domain, which is found in a variety of bacterial and phage surface proteins involved in bacterial host-cell interaction [33]. The tail module of BH1 (ORF12 to ORF16) is highly related to the genome sequence generated for phage Lrm1. This cluster also bears resemblance in both gene organization and nucleotide sequences to the tail morphogenic region of J-1, PL-1, and A2 (Dataset S1). However, BH1 ORF15, encoding a phage tail component, shares high homology only with N-terminus of Lrm1 ORF14 (~310 aa). Interestingly, the second half of ORF15 exhibits high similarity with the C-terminus of the corresponding protein described for bacteriophage Lc-Nu (~385 aa). Such a mosaic structure was also observed in the next annotated gene (ORF16), which is probably associated with endopeptidase activity. Notably, another two genes (ORF17 and ORF18) with an unknown function are not present in the genome of Lrm1. However, they display a high level of nucleotide sequence identity (99.7% and 96.5%, respectively) with adequate genes of phage Lc-Nu. Additionally, ORF19 was not found either in the genome of Lrm1 and also Lc-Nu, but was nearly 100% identical with sequences determined for bacteriophage A2 and iLp84 (Dataset S1). These results suggest that BH1 has a chimeric character, and may be derived from different ancestors [34,35].

The lysis module consists of two genes, and resembles the structure reported for many prophages in the *L. casei* group (Dataset S2, S3, S4). ORF20 codes for holin, an enzyme which causes bacterial membrane lysis [36]. The next gene, which typically encodes lysin, is conserved between *L. rhamnosus* phages (>90% nt identity) (Dataset S1). Similar to other previously described bacteriophages of lactobacilli, this enzyme is endolysin. Endolysin exhibits 1,4-beta-*N*-acetylmuramidase activity, and by hydrolysis of the amide bond in peptidoglycan, participates in bacterial cell wall degradation [37]. Typically, lysins have two LysM domains on the C-terminus and Glyco-25 motif on N-terminus. It has also been shown that the analyzed protein contains a signal peptide. For BH1, the predicted cleavage site was identified between position 28 and 29 of the amino acid sequence (probability of 0.959).

The next coding region (ORF22) of BH1 encodes protein of unknown function, and was observed only for *L. paracasei* phage iLp84 (coverage 100%, identity 99%) and *L. casei* phage Lca1 (coverage 96%, identity 96%). Comparative analysis showed that the sequence between ORF22 and ORF23 contains the attachment region (*att*P) of 90 bp, with a putative core site 15 nucleotides in length. Based on the complete genome sequence of the bacterial host and results obtained using Sanger sequencing, the *att*B, *att*L, and *att*R regions were also determined (Figure 3). This allowed for accurate identification of the location of prophage sequence in the genome of *L. rhamnosus* Pen. BLASTn comparison of *att*B sequence generated for *L. rhamnosus* Pen with genome sequences of other *L. rhamnosus* isolates revealed that many bacterial strains possess an empty integration region, which is located between tRNA^ser^ and tRNA^val^ genes. Additionally, similar *att*P sequence was also observed in the genomes of phage Lrm1 and Lc-Nu [14,15]. It was typical that the core of *att*B was located within the tRNA ser gene (close to 3′ end) and presumably phage integration does not affect on tRNA integrity [29,38]. It is also noteworthy that the location of prophage BH1 is slightly different in comparison to most of the prophages that possess a similar attachment site. In the case of BH1, we observed an additional ORF encoding bacterial integrase between the typical tRNA^val^ gene and *att*L sequence. Interestingly, this gene appears in six copies in the genome of *L. rhamnosus* Pen.

Open reading frame (ORF 23)—localized adjacent to *att*P—codes for integrase, an enzyme which catalyses site-specific recombination between the integration site of bacteriophage, and those located in the bacterial genomes. Among all phages described for the *L. casei* group, a similar gene for integrase was only detected for Lrm1 (98.5% identity). Analysis of the amino acid sequence of BH1 integrase revealed the presence of several distinctive protein domains, which are likely to bind DNA and cause recombination. On this basis, this enzyme can be classified as tyrosine integrases, which, unlike serine recombinases, utilize tyrosine to mediate DNA cleavage, recognize definitely longer *att*P sequences, and require host cofactors [39].

For the sequences of the next four genes, from ORF24 to ORF27, we detected no similarity to the previously described phages for *L. casei* group. Nevertheless, similar or even identical sequences have been identified in many genome sequences of *L. rhamnosus*, *L.casei,* and *L. paracasei*, in presumably prophage regions. The analysis of amino acid sequences of proteins encoded by ORF24 and ORF25 did not indicate the presence of characteristic protein domains. As a result, it is difficult to make conclusions about the presumable functions of these proteins. The ORF24 sequence is usually located in the immediate vicinity of the gene encoding the phage integrase. Since the orientation of these two genes is also the same, we assume that the protein encoded by this gene is important for the integration of phage DNA within the bacterial chromosome. Furthermore, an integrase sequence similar to ORF23 of the phage BH1 has been identified in 81 strains of *L. rhamnosus,* and for most of these strains (74) an accompanying gene (similar to ORF24) has also been detected. The presence of a gene similar to ORF24 has not been observed when there was no sequence encoding the integrase (Dataset S2). The amino acid sequence of the protein encoded by ORF26 has conserved domains characteristic for restriction endonuclease subunit S. Interestingly, as well as the sequences located within the genomes of phages and prophages, a similar sequence was also observed within the genomes of many bacteria, not only of the genus *Lactobacillus*. In this case, these genes were part of the type I restriction-modification system, typically composed of three subunits: Restriction (HsdR) subunit, HsdM modification (M) subunit, and HsdS recognition (S for specificity) subunit [40,41]. Preliminary analysis of the ORF27 sequence did not indicate the function of this gene. However, using the conserved domains database (NCBI), in the amino acid sequence, protein domains characteristic for peptidase S24 LexA-like proteins were identified. These are involved in the SOS response, leading to the repair of single-stranded DNA [42]. It has also been shown that these proteins can indirectly influence the level of spontaneous prophage induction [17,43,44]. The next gene, similar to ORF27, is also transcribed leftward, and the protein encoded by this ORF presumably regulates the transcription of genes when there is cellular stress associated with DNA damage, which may be related to the genetic switch of phages from a lysogenic pathway to a lytic pathway [42,45]. A similar sequence has also been observed in the genomes of J-1 phages (coverage 69%, identity 87%) and phiAT3 (coverage 100%, identity 91%). Despite its function similar to that of ORF28, that is, to regulate the gene transcription (XRE family transcriptional regulator), ORF29 was homologous to two other bacteriophages, namely C_L_1 and C_L_2. This is another example of the immense variability of bacteriophages described for the *L. casei* group.

Identifying the function of the genes encoded in the next portion of the bacteriophage BH1 genome (ORF30–ORF40) proved challenging. For example, the next two genes (ORF30 and 31) encode short-chain proteins with an unknown function (hypothetical protein). Interestingly, similar genes were not observed in the *L. casei* group bacteriophages described so far (Dataset S1). Illustrating the mosaic nature of the bacteriophage genome, subsequent genes were identified in sequences of different phages, e.g., Lrm1 (ORF32–ORF41), phiAT3 (ORF32–ORF40), J-1 (ORF32–ORF35), PL-1 (ORF32–ORF35), Lc-Nu (ORF: 34, 35, 37, 38), C_L_2 (ORF: 33, 34, 37), A2 (ORF: 34, 37), C_L_1 (ORF33, ORF34), and Lca1 (ORF40). For bacteriophage Lrm1, a region very similar to the sequence located between ORF32 and ORF39 has been described as a DNA replication module [15]. However, since in most cases there are no characteristic protein domains, the molecular function of individual genes seems to be, at most, hypothetically determinable (Table 3).

Comparative analysis has shown that a sequence very similar to ORF34 is present in genomes of many phages and prophages. Based on the similarity to *S. aureus* and *S. epidermidis*, we suggest that the product of this gene may act as a transposase, however, is likely nonfunctional [22]. Interestingly, the protein encoded by ORF36 has the domain Sipho_Gp157 (pfam05565), the presence of which is associated with bacterial resistance to bacteriophages [46]. Another gene encodes a phage-derived protein with an unknown function, often found among phage proteins of Gram-positive bacteria. These proteins usually contain characteristic P-loop motifs (G/A-X-X-G-X-G-K-T), which are located at their N-termini. The amino acid sequences of proteins encoded by ORF37–ORF40 implies that the analysed region of phage BH1 is associated with DNA replication, recombination, and repair. This is supported by the identification of protein domains characteristic for phage nucleotide-binding protein (ORF37), single-stranded DNA-binding protein (ORF38), IstB-like ATP binding protein (ORF40), DNA replication protein DnaC (ORF40), and Holliday junction DNA helicase (ORF40).

The final portion of the phage BH1 was found to be even further differentiated. A few genes are characteristic only for the phage BH1, e.g., ORF50, ORF52, and ORF53, as well as ORF45 and ORF49 (excluding the phage Lc-Nu). Functional analysis has shown that some of the examined genes can encode protein components of restriction-modification systems, such as endonucleases of ORF43, ORF44, and ORF55, etc. or methylases of ORF45. In the case of ORF50–ORF54, based on existing domains, the protein products of these genes are likely to control the expression of viral genes as part of the lysogeny/lytic growth switch. Interestingly, ORF56 encodes a protein that contains domains specific to Glutaredoxin-like protein NrdH, which may serve as a hydrogen donor for the ribonucleotide reductase during deoxyribonucleotide biosynthesis [47].

### 3.4. Spontaneous Phage Induction Analysis Using QPCR and Digital PCR

The final purpose of our study was the quantitative analysis of spontaneous induction of bacteriophage BH1, using the qPCR and digital PCR droplet techniques. The designed testing system included the quantitative analysis of sequences of *att*B (level of re-established *att*B sites in bacterial genome), *att*P (level of extrachromosomal phage DNA), *att*L and *att*R (amount of integrated prophages in the bacterial chromosome), in comparison to the reference gene originating from the genome of *L. rhamnosus* bacteria. This method has been previously described by Lunde et al. and is particularly useful in cases where the plate tests aimed to quantify the bacteriophage do not give positive results (lack of suitable indicator strain) [48]. Based on the genome sequence of the phage BH1 and genome of the *L. rhamnosus* Pen, five pairs of primers were designed and used in qPCR and ddPCR.

**Figure 5:**
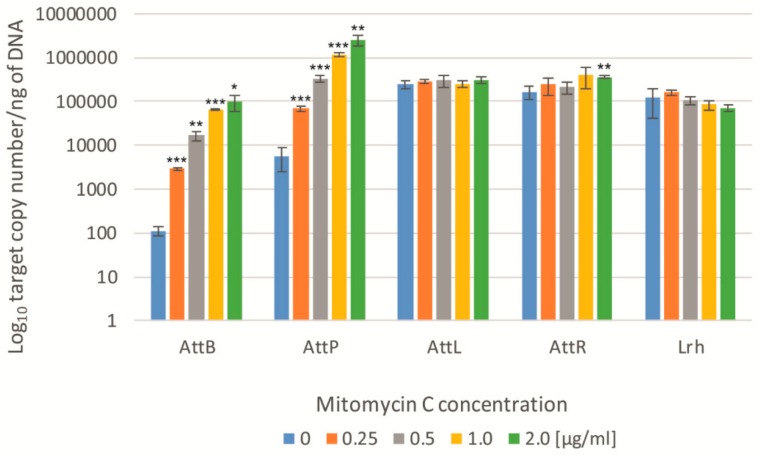
Droplet digital PCR analysis of phage BH1 induction in the presence of different concentrations of mitomycin C. Induction level was observed by measuring the amount of phage attachment sequences (*att*B, *att*P, *att*L, and *att*R). Additionally, the quantity of bacterial DNA (Lrh) was also monitored. The *L. rhamnosus* cultures were induced using increasing amounts of mitomycin C; 0.25 µg/mL—orange bars, 0.5 µg/mL—gray bars, 1 µg/mL—yellow bars, and 2 µg/mL—green bars. The obtained results were analyzed according to the control culture (without inducing agent—blue bars). Error bars show the standard deviations of the means. *p < 0.05, **p < 0.01, ***p < 0.001.

Cells of *L. rhamnosus* Pen were exposed to mitomycin C for 6 h, and DNA isolated. The results showed a significant increase in the amount of a circular phage DNA, with an increase in the concentration of mitomycin (*att*P system) (Figure 5 and Figure S1A). Even at the lowest concentration (0.25 µg/mL), the *att*P/Lrh ratio increased more than 5-fold at 6 h after the induction. For the highest concentration of mitomycin, the *att*P/Lrh ratio increased more than 300-fold compared to the control sample. The ddPCR analysis of the *att*P absolute value has shown that it increased 12-fold and, in the other case, over 400-fold (for mitomycin concentrations of 0.25 and 2 µg/mL, respectively). The frequency of induction/excision of prophage BH1 was also observed using the *att*B/Lrh ratio, which corresponded to the proportion of bacteria with the re-established chromosome (after prophage excision). For this experimental system, the *att*B/Lrh ratio increased more than 8-fold for the mitomycin concentration of 0.25, and over 230-fold for its concentration of 2 µg/mL (Figure S1B). The results obtained in the ddPCR show a 25-fold and over 850-fold increase, respectively, in comparison to the control sample that was not induced. These results and previous studies suggest that the ddPCR method, compared to qPCR, has a significantly higher sensitivity, especially in experiments aiming to detect a small number of DNA matrix copies [49,50]. As in the case of studies by Lunde et al. [48], for experiments in which the amount of integrated prophages *att*L and *att*R was measured, no significant differences in the *att*L/Lrh and *att*P/Lrh ratio were obtained for samples treated with increasing concentrations of mitomycin (data not shown). Similar results were also obtained using the ddPCR method (Figure 5). Since the absolute number of lysogenic bacterial cells is similar to the total number of bacteria in the tested samples (even under induced conditions), primer systems designed for *att*L and *att*P have proven to be unsuitable for observing the induction of prophage sequences [48].

In summary, our results demonstrate that the designed primer sets for *att*P and *att*B allow the observation of the prophage BH1 induction. However, it should be stressed that the number of *att*P copies may be affected by the replication of circular phage particles, as well as by the gradual lysis and release of mature phage particles from induced cells. These processes can be monitored by analysing the changes in the *att*P/*att*B ratio. When evaluating the amount of cells that have been induced, the observation of the amount of copies of *att*B seems to be the most reliable approach. However, the lysis of cells, which occurs as a result of induction, may affect the results obtained. It is also worth noting that, especially in terms of monitoring the *att*B copy count, ddPCR has proven to be a much more sensitive method, enabling the precise detection of even a small number of copies of examined DNA molecules [49].

**Figure 6:**
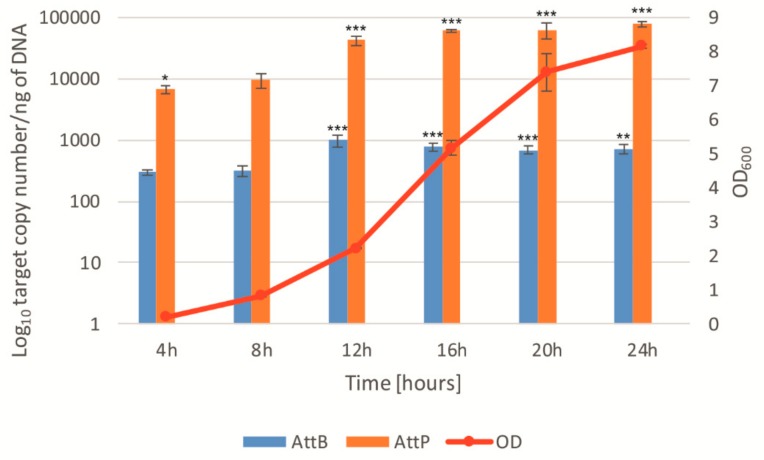
Analysis of spontaneous prophage induction during *L. rhamnosus* Pen growth using droplet digital PCR. SPI was monitored by measuring excision events (*att*B—blue bars) and level of extrachromosomal phage DNA (*att*P). Bacterial culture growth was observed based on OD_600_ measurements (red line). Error bars show the standard deviations of the means. * *p* < 0.05, ** *p* < 0.01, *** *p <* 0.001.

We also analysed the induction of the prophage BH1 during the growth of the bacterial host, expecting a significantly lower level of induction. ddPCR was used to monitor the number of copies of *att*P, *att*B, and the reference gene (bacterial chromosomal gene) during the culture of *L. rhamnosus* Pen. Measurements were taken every 4 h during a 24 h incubation at 37 °C. Analysis of the results showed a gradual increase in the number of copies of the *att*P and *att*B sequences during the bacterial culture, in the absence of any known inducer (Figure 6). In the case of *att*B, there was an almost 2.5-fold increase in the amount of the sequences resulting from the release of the prophage from the bacterial genome at the 4 and 24 h samples. For the *att*P sequence, the increase was almost 12-fold (similar results were obtained with the qPCR method—Figure S2). In both cases, a significant increase in induction was observed between 8 and 12 h of the culture, for the mid-exponential growth phase of tested bacteria. These results unambiguously confirmed previous microscopic observations of *L. rhamnosus* Pen releasing bacteriophage particles during growth. This release was spontaneous, and did not result from the use of inductive factors [16].

Mechanistically, the factors causing spontaneous induction, as well as the multi-faceted significance of this process, have been frequently analysed. Previous studies indicate that the phenomenon of spontaneous lysogen induction may be caused by extracellular stress related to UV radiation, reactive oxygen species (ROS), temperature, and pH changes. Intracellular factors may also cause a subpopulation of cells to start induction and release phage particles. These triggers include stalled replication forks, reactive oxygen species, and noise in gene expression [17]. From the perspective of technological processes using living microbes for the production of fermented food, spontaneous induction of prophages is unfavourable. When lysis takes place in starter strains used in fermentation processes, or for probiotic strains used in production of protective drugs, large financial losses occur. However, it should also be emphasized that many bacteriophages have a temperate nature, and for this reason, their induction does not have to lead to total lysis and the obstruction of fermentation. It is also worth noting that spontaneous phage induction is likely a natural process, which may take on a unique nature depending on the kind of bacteriophage and bacterial strain, giving unique characteristics to the products being fermented [11,15,51,52].

By analysing the spontaneous induction from the “perspective” of bacteria, it can be concluded from previous studies that this process may be important for a given strain and for the whole ecological niche [12]. Studies have shown that prophages that can be induced spontaneously have a positive effect on the general fitness of bacterial population under diverse environmental conditions [13]. It has been demonstrated that phage induction plays an important role in the formation of biofilm, superinfection exclusion, and in shaping the equilibrium of a specific bacterial community [12,17,53]. Furthermore, the spontaneous induction appears also to be important in building the genetic variability of bacteriophages and bacterial hosts. Baugher et al. have shown that SPI may lead to a horizontal gene transfer, thus giving microbes new characteristics that can give them an advantage in the competition for an ecological niche [54]. It is also worth considering the extreme variability present in the genetic material of the bacteriophages themselves [55]. Bacteriophages, prophages, and prophage-like elements are considered to be the so-called dark matter of biodiversity [34]. It appears that the reason for such an astounding variability of bacteriophages is the high virus mobility resulting from the spontaneous prophage induction, underestimated in its function. Compared to a very “spectacular” lytic cycle, only a small subpopulation of bacteria undergoes SPI, thereby releasing a relatively small number of phage particles. However, by scaling this process up to the level of an entire ecological niche, the importance of SPI in shaping biodiversity of microbial and phage communities appears to be substantial. It has also been demonstrated that many environmental factors can enhance the SPI, potentially contributing to an increase in the number of free bacteriophages in the natural environment, causing phages to have even a greater impact on the ecological niche [52,56].

Finally, SPI has an important role in bacterial virulence. Studies have demonstrated that prophage induction may indirectly contribute to the release of bacterial toxins, and increase the ability of bacteria to adhere to host cells [17,57,58]. It has also been shown that by facilitating binding to human platelets, SPI may lead to development of infective endocarditis [59,60]. It is therefore important to ask the question of whether spontaneous prophage activity promotes host virulence—not only in the case of bacteria commonly considered to be pathogenic—but also in the case of probiotic microorganisms, which are a rich reservoir of prophage sequences [25]. There are numerous cases of bacteremia and endocarditis caused by probiotic bacteria belonging to the species *Lactobacillus rhamnosus* in the literature [61,62,63]. An example is the *L. rhamnosus* Pen, which has many documented pro-health properties, but has also been associated with sepsis in a cardiosurgical patient [3,64]. Importantly, in relation to the resistance to many antibiotics, the treatment of infections caused by lactobacilli can be challenging [65]. Therefore, it is crucial that further research is conducted in order to identify potential determinants that may be essential for the development of bacteremia and infective endocarditis. Future research should cover not only typically pathogenic microbes, but also probiotic strains used for the production of protective medications and those used as food additives. We suggest that prophage sequences in their genomes and spontaneous prophage induction represent important factors promoting the virulence of these bacteria.

## 4. Conclusions

To sum up, a thorough insight into the genome phages and prophages of lactobacilli belonging to the *L. casei* group showed that these mobile elements are genetically extremely diverse. Moreover, spontaneous induction of ϕBH1 was also observed using qPCR and ddPCR, and interestingly, this phenomenon did not significantly affect the growth of bacterial culture. Despite the lack of direct evidence on the physiological significance of this process for the tested strain, it has been previously suggested that SPI may enhance biofilm formation and lead to horizontal gene transfer. On the other hand, by facilitating binding to human platelets, SPI may also play an important role in bacterial virulence. Nevertheless, further research is needed to confirm both the positive effects of SPI and the potential risks associated with prophages and their spontaneous induction. This will not only allow us to learn the biological significance of the spontaneous process of prophage induction, but can also contribute to a more conscious use of living microorganisms in the industry. Additionally, we hope that the obtained results and further research will also lead to the extension of knowledge regarding interactions between phages and bacteria, which—excluding the classical lytic cycle—seems to be still insufficient.

## Figures and Tables

**Table 1:** PCR primers used in qPCR and droplet digital (dd)PCR reactions.

Primer Set	Amplified Fragment	Primer Sequences (5′–3′)	Position	GenBank Accession Number
1	*att*B	AGGTCTCAATTTGGTCTCAAAA	2237443–2237464	CP020464.1
		ACGGGTTTTGCCTGTATCAA	2278301–2278320	CP020464.1
2	*att*R	ATTTTGTGCACCGGAGGAA	21564–21582 2278166–2278184	MH983004.1 CP020464.1
		ACGGGTTTTGCCTGTATCAA	2278301–2278320	CP020464.1
3	*att*L	AGGTCTCAATTTGGTCTCAAAA	2237443–2237464	CP020464.1
		AAATATGCACAAAATGTGGCTTT	21408–21430 2237597–2237619	MH983004.1 CP020464.1
4	*att*P	TGTGCACCGGAGGAAAAA	21561–21578 2278170–2278187	MH983004.1 CP020464.1
		AAATATGCACAAAATGTGGCTTT	21408–21430 2237597–2237619	MH983004.1 CP020464.1
5	Lrh	TTGACAAGGGACTCAAGGAT	2208780–2208799	CP020464.1
		TATGATAGCCGGAATCAGCA	2208910–2208929	CP020464.1

**Table 2:** Identification of bacteriophage-derived proteins using LC–MS/MS procedure.

Protein Description	Mascot Score ^a^	No. of Unique Peptides Matched ^b^	Protein Coverage [% aa]	Protein Molecular Mass	Orf
phage tail tape measure protein [Lactobacillus rhamnosus]	1978	28	19.4	173159	14
phage tail protein [Lactobacillus rhamnosus]	2851	35	37.4	107736	16
phage tail protein [Lactobacillus rhamnosus]	1375	18	32.4	71309	15
phage portal protein [Lactobacillus rhamnosus]	1214	16	47.7	46346	4
major capsid protein [Lactobacillus rhamnosus]	2371	25	78	43998	6
hypothetical protein [Lactobacillus rhamnosus]	192	2	49.4	7776	7
phage tail protein [Lactobacillus rhamnosus]	1345	16	75.1	22056	12
phage tail protein [Lactobacillus rhamnosus]	755	11	95.3	14653	11
phage head-tail adapter protein [Lactobacillus rhamnosus]	388	7	58.7	12598	9
DNA-packaging protein [Lactobacillus rhamnosus]	439	7	47.9	13581	8
hypothetical protein [Lactobacillus rhamnosus]	1239	17	72.7	17915	36
phage holin [Lactobacillus rhamnosus]	120	2	6.6	14373	20
peptidase U35 [Lactobacillus rhamnosus]	37	1	5.7	23706	5
hypothetical protein [Lactobacillus rhamnosus]	234	4	31.2	14024	10
transcriptional regulator [Lactobacillus rhamnosus]	49	1	8.4	9473	29
hypothetical protein [Lactobacillus rhamnosus]	43	1	21.7	6948	46
hypothetical protein [Lactobacillus rhamnosus]	252	4	36.4	7358	31

^a^ Protein scores derived from individual ion scores of Mascot-identified tryptic peptides as a nonprobabilistic basis for ranking protein hits. ^b^ Total number of tryptic peptides which the Mascot program assigned to a database protein. To compute this number, multiple matches to lactobacillar peptides with the same primary sequence but representing different charge or modification states were counted as one.

**Table 3:** General features of putative ORFs of bacteriophage BH1 and nucleotide sequence homology with genome sequences of phages Lrm1 and Lc-Nu.

Orf Order/Strand	Predicted Start Stop Site	GC (%)	Orf Length (aa)	Predicted Molecular Mass (kDa)	pI	Description	Representative orf (% Coverage/% nt Identity)		Biological Process/Molecular Function ^a^
							Lrm1 NC_011104.1	Lc-Nu NC_007501.1	
1F	90–545	41.01	151	17.06	8.85	Terminase, small subunit	100/99.34	0/0	-
2F	567–2279	43.96	570	65.10	5.15	Terminase, large subunit	100/93.35	0/0	-
3F	2291–2482	42.19	63	6.66	9.31	Hypothetical protein	100/98.44	0/0	-
4F	2488–3741	46.65	417	46.32	5.07	Portal protein	100/81.64	0/0	-
5F	3695–4324	47.62	209	23.72	5.45	Prohead protease	100/87.78	0/0	-
6F	4366–5568	46.22	400	44.03	5.42	Major capsid protein	98.92/ 84.37	0/0	-
7F	5586–5825	50.83	79	77.63	4.53	Hypothetical protein	0/0	0/0	GO:0030246
8F	5836–6195	41.94	119	13.52	4.24	Putative DNA-packaging protein	46.67/91.67	0/0	-
9F	6185–6514	47.58	109	12.61	9.57	Head-tail adaptor	100/98.48	0/0	-
10F	6514–6900	47.80	128	14.03	5.53	Putative tail component	100/96.12	0/0	-
11F	6900–7286	45.22	128	14.66	4.39	Putative head-to-tail joining protein	100/95.61	0/0	-
12F	7320–7937	44.82	205	22.07	4.56	Major tail protein	96.12/95.79	0/0	-
13F	8036–8449	42.51	137	15.24	6.30	Hypothetical protein	100/98.31	0/0	-
14F	8572–13434	45.86	1620	173.27	9.42	Tail tape measure protein	97.27/95.48	0/0	-
15F	13435–15351	46.69	638	71.17	5.21	Tail component protein	47.99/89.67	59.36/82.86	-
16F	15352–18306	45.69	984	107.75	4.78	Prophage tail endopeptidase	36.75/82.60	55.40/79.54	-
17F	18322–18651	45.76	109	11.90	4.38	Hypothetical protein	0/0	100/99.70	-
18F	18648–18791	42.36	47	5.29	4.86	Uncharacterized protein, XkdX family	0/0	100/96.53	-
19F	18823–19122	45.33	99	11.30	6.63	Hypothetical protein	0/0	0/0	-
20F	19137–19550	49.76	137	14.38	4.82	Holin	0/0	0/0	-
21F	19561–20859	48.11	432	47.06	8.98	Glycoside hydrolase, lysin	100/90.87	100/92.33	GO:0009253 GO:0016998 GO:0003796
22F	20904–21128	45.78	74	8.18	4.97	Hypothetical protein	0/0	0/0	-
23R	21592–22719	42.55	375	43.28	9.71	Site-specific integrase	100/98.49	0/0	GO:0006310 GO:0015074 GO:0003677
24R	22827–23090	35.23	87	10.24	9.80	Hypothetical protein	0/0	0/0	-
25F	23229–23450	42.34	73	8.13	4.32	Hypothetical protein	0/0	0/0	-
26R	23560–24741	40.78	393	44.33	9.00	Restriction endonuclease, type I, HsdS	0/0	0/0	GO:0006304 GO:0003677
27R	24832–25050	47.03	72	8.07	6.53	LexA-like peptidase	0/0	0/0	-
28R	25122–25895	42.77	257	28.97	4.61	Cro/CI-type transcriptional repressor	0/0	0/0	GO:0003677
29F	26053–26304	39.29	83	9.48	10.2	XRE family transcriptional regulator	0/0	0/0	GO:0003677
30F	26301–26450	44.00	49	5.45	6.12	Hypothetical protein	0/0	0/0	-
31R	26447–26647	37.81	66	7.30	5.33	Hypothetical protein	0/0	0/0	-
32F	26722–27078	42.58	118	13.92	9.15	DUF771 domain-containing protein	100/100	0/0	-
33F	27078–27170	47.31	30	3.36	5.96	Hypothetical protein	100/97.85	0/0	-
34F	27163–27315	45.10	50	5.69	9.52	Hypothetical protein	100/95.42	98.69/96.69	-
35F	27320–27523	45.59	67	7.51	6.00	Hypothetical protein	100/86.27	100/90.20	-
36F	27542–28027	46.30	161	17.92	8.73	Siphovirus-like Gp157 protein	100/96.91	0/0	-
37F	28028–28735	45.34	235	26.57	6.77	Nucleotide-binding protein	100/94.92	100/92.95	-
38F	28739–29296	43.37	185	20.92	5.50	DUF669 domain-containing protein	100/95.17	97.13/96.13	-
39F	29311–30108	43.86	265	31.13	9.36	Putative replication protein	41.73/85.29	0/0	-
40F	30095–30877	46.87	260	29.73	9.68	IstB-like ATP binding protein	100/86.75	0/0	GO:0005524
41F	30874–31218	43.77	114	13.09	4.51	Hypothetical protein	94.49/ 93.56	0/0	-
42F	31205–31459	50.20	84	9.42	9.75	Hypothetical protein	0/0	100/90.59	-
43F	31456–31821	43.71	121	14.39	6.92	Holliday junction resolvase RusA-like	0/0	0/0	GO:0006281 GO:0006310 GO:0000287
44F	31832–32170	46.02	112	12.44	5.38	Putative endonuclease	0/0	0/0	-
45F	32182–32883	45.44	233	27.11	5.01	SAM-dependent DNA methyltransferase	0/0	96.15/93.63	GO:0006306 GO:0003677 GO:0008170
46F	32880–33062	41.53	60	6.89	4.55	Hypothetical protein	55.19/94.06	100/94.54	-
47F	33059–33601	49.35	180	20.64	4.85	DUF1642 domain-containing protein	8.47/93.48	0/0	-
48F	33757–34137	45.41	126	14.21	4.40	Hypothetical protein	10.24/97.44	0/0	-
49F	34134–34343	40.95	69	8.34	6.73	Hypothetical protein	0/0	100/ 94.79	-
50F	34471–34692	42.34	73	8.42	9.52	HTH-transcriptional regulator	0/0	0/0	GO:0003677
51F	34762–35205	47.97	147	16.85	7.88	Transcriptional regulator, ArpU family	55.86/ 97.98	55.63/97.57	-
52F	35599–36672	35.85	357	40.65	6.33	Hypothetical protein	0/0	0/0	-
53R	36834–37133	42.00	99	10.81	9.37	Stress response protein CsbD	0/0	0/0	-
54F	37552–38769	45.07	405	46.64	6.07	GcrA cell cycle regulator	0/0	100/95.81	-
55F	38717–39286	44.91	189	21.44	9.28	HNH endonuclease	0/0	22.81/84.62	GO:0016788
56F	39290–39613	46.91	107	12.69	9.01	Ribonucleoside-diphosphate reductase	81.48/78.79	80.56/89.27	-
57F	39816–40610	45.28	264	31.14	9.05	Small terminase subunit/HNH endonuclease	100/97.99	0/0	GO:0003676 GO:0004519

^a^ Biological process and molecular function of proteins was determined using InterProScan.

## Supplementary Materials

Supplementary materials can be found at https://www.mdpi.com/1999-4915/11/12/1163/s1. Figure S1: qPCR analysis of phage BH1 induction in the presence of different concentrations of mitomycin C. Induction was observed by measuring the relative amount of extrachromosomal phage DNA (*att*P) (**A**) and the level of reestablished *att*B sites in bacterial genome (**B**). * *p* < 0.05, ** *p* < 0.01, *** *p <* 0.001. Figure S2: Relative level of extrachromosomal phage DNA (*att*P) during growth of *L. rhamnosus* Pen monitored using Q-PCR method. * *p* < 0.05, ** *p* < 0.01, *** *p* < 0.001. Dataset S1: Comparison of coding sequences of bacteriophage BH1 with genome sequences of phages described for *Lactobacillus casei* group. Dataset S2: Comparison of coding sequences of bacteriophage BH1 with prophage sequences derived from *Lactobacillus rhamnosus* strains. Dataset S3: Comparison of coding sequences of bacteriophage BH1 with prophage sequences derived from *Lactobacillus casei* strains. Dataset S4: Comparison of coding sequences of bacteriophage BH1 with prophage sequences derived from *Lactobacillus paracasei* strains.
