# Supplementary figures and images for: Genomic and Proteomic Characterization of Bacteriophage BH1 Spontaneously Released from Probiotic Lactobacillus rhamnosus Pen
Source: Viruses. 2019 Dec 16;11(12):1163. doi: 10.3390/v11121163 (PMC6950654; doi:10.3390/v11121163)

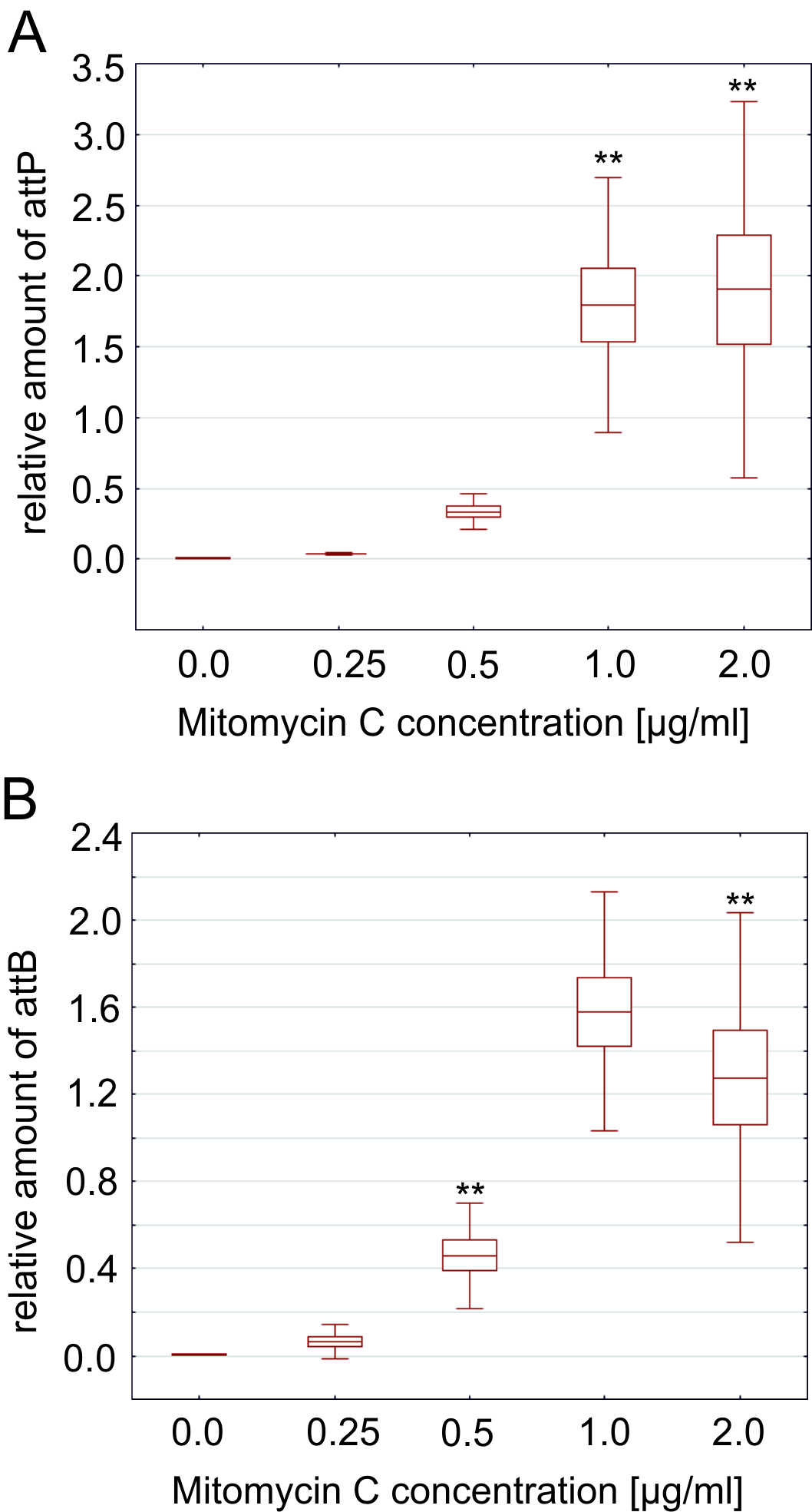

Supplement: Supplementary file 1 [file viruses-11-01163-s001.zip › Figure S1.tif]

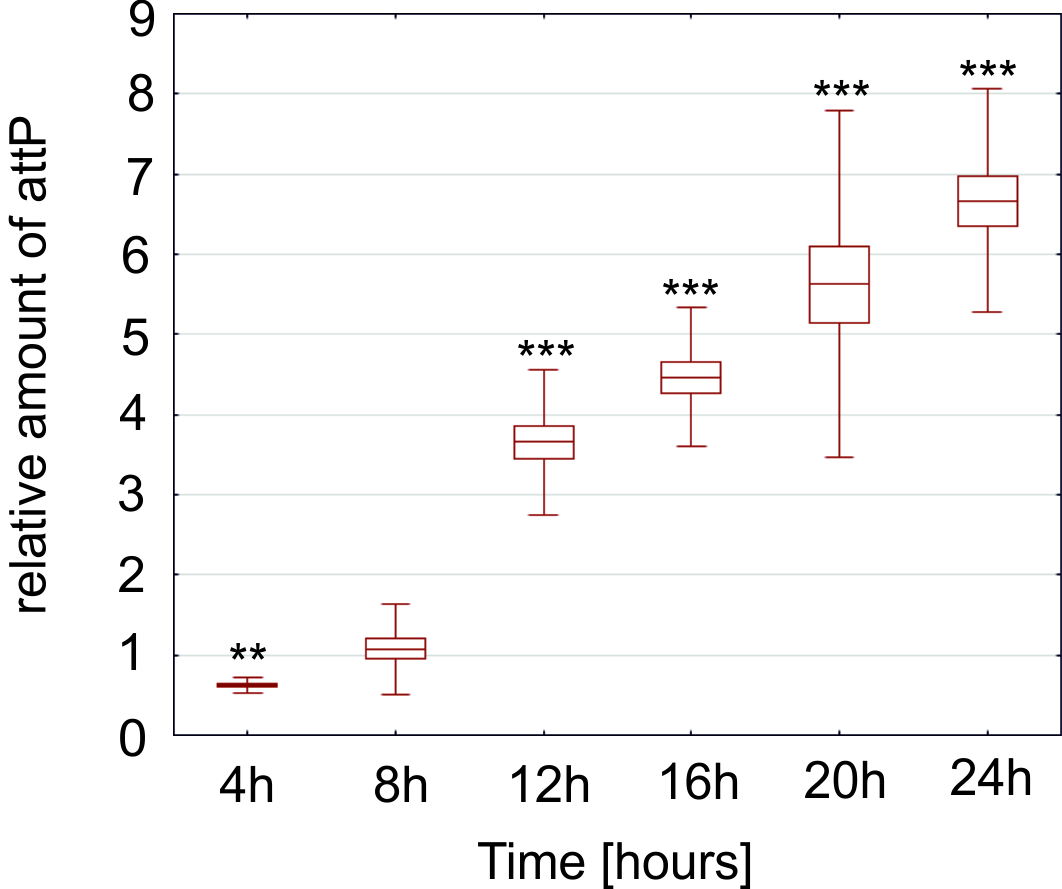

Supplement: Supplementary file 1 [file viruses-11-01163-s001.zip › Figure S2.tif]
